# Sensitivity of IFN-γ Release Assay to Detect Latent Tuberculosis Infection Is Retained in HIV-Infected Patients but Dependent on HIV/AIDS Progression

**DOI:** 10.1371/journal.pone.0001441

**Published:** 2008-01-16

**Authors:** Farba Karam, Fatou Mbow, Helen Fletcher, Cheikh S. Senghor, Koura D. Coulibaly, Andrea M. LeFevre, Ndeye F. Ngom Gueye, Tandakha Dieye, Papa S. Sow, Souleymane Mboup, Christian Lienhardt

**Affiliations:** 1 Laboratoire de Bactériologie – Virologie, Hôpital Aristide Le Dantec, Dakar, Senegal; 2 Institut de Recherche pour le Développement, Programme Tuberculose, Dakar, Senegal; 3 Centre for Clinical Vaccinology and Tropical Medicine, Churchill Hospital, University of Oxford, Oxford, United Kingdom; 4 Clinique des Maladies Infectieuses, Hopital Fann, Dakar, Senegal; 5 Infectious Disease Epidemiology Unit, London School of Hygiene and Tropical Medicine, London, United Kingdom; 6 Centre de Traitement Ambulatoire, Hopital Fann, Dakar, Senegal; 7 Clinical Trial Division, International Union Against Tuberculosis and Lung Diseases, Paris, France; National AIDS Research Institute, India

## Abstract

**Background:**

Detection and treatment of latent TB infection (LTBI) in HIV infected individuals is strongly recommended to decrease morbidity and mortality in countries with high levels of HIV.

**Objective:**

To assess the validity of a newly developed in-house ELISPOT interferon-γ release assay (IGRA) for the detection of LTBI amongst HIV infected individuals, in comparison with the Tuberculin Skin Test (TST).

**Methodology/Principal Findings:**

ESAT6/CFP10 (EC) ELISPOT assays were performed, together with a TST, in 285 HIV infected individuals recruited in HIV clinics in Dakar, Senegal, who had no signs of active TB at time of enrolment. Thirty eight of the subjects (13.3%) failed to respond to PHA stimulation and were excluded from the analysis. In the 247 remaining patients, response to PHA did not vary according to CD4 cell count categories (p = 0.51). EC ELISPOT was positive in 125 (50.6%) subjects, while 53 (21.5%) had a positive TST. Concordance between EC ELISPOT and TST was observed in 151 patients (61.1%) (kappa = 0.23). The proportion of subjects with a positive response to the EC ELISPOT assay decreased with declining CD4 counts (p trend = 0.001), but were consistently higher than the proportion of TST responders. In multivariate analysis, the risk of being EC-ELISPOT positive in HIV infected individuals was associated with age, CD4 count and HIV-1 strain.

**Conclusion:**

Our study indicates that IGRAs using *M. tuberculosis* specific antigens are likely to retain their validity for the diagnosis of LTBI among HIV positive individuals, but may be impaired by T-cell anergy in severely immuno-suppressed individuals.

## Introduction

Tuberculosis (TB) kills more than two million people each year and is one of the world's leading causes of death due to infection among young people and adults [1]. Due to the combined effects of economic instability, the breakdown of health systems, the spread of HIV/AIDS and the emergence of multi-drug resistant forms, the TB burden is increasing in many resource-poor countries. HIV/AIDS and TB have a deadly synergy, as HIV infection promotes progression from TB infection to disease [2] and TB accelerates the course of HIV disease [3]. A recent study in gold-miners in South Africa showed that the incidence of TB disease doubled within the first year of HIV seroconversion [4]. Chemoprophylaxis of TB in HIV infected persons is strongly recommended [5] but the identification of TB infection in those individuals is difficult. For many years, latent TB infection (LTBI) has been identified using the Tuberculin Skin Test (TST), which measures a delayed-type hypersensitivity response to a purified protein derivative (PPD) of more than 200 *M. tuberculosis* (MTB) antigens [6]. Despite its widespread use, TST unfortunately suffers major limitations due to cross-reactions with a wide range of environmental mycobacteria and BCG vaccination, and its sensitivity has been shown to be substantially reduced in HIV-infected individuals [7].

The characterisation of immunogenic antigens in the Region of Difference 1 (RD-1), a genomic region present in the *M. tuberculosis* complex but deleted from *M. bovis* BCG and most environmental mycobacteria, has allowed the development of highly-specific immuno-diagnostic tests for TB infection [8]. In particular, strong immune responses to the ESAT-6 and CFP-10 antigens have been shown to correlate with TB infection, even in asymptomatic individuals, and several interferon-γ release assays (IGRA) have been developed using these specific antigens [9], [10], [11]. A number of studies have showed that IGRAs using ESAT-6/CFP-10 (EC) antigens were more specific than the TST for the diagnosis of latent TB infection in endemic settings [11], [12]. There is however limited evidence of the value of these assays among immuno-compromised individuals, such as those with HIV-infection [13], [14]. The importance of the interaction between HIV and TB urges us to develop a highly sensitive and specific test for the detection of LTBI amongst HIV positive patients. The objective of this study was to investigate, in a high TB prevalent area, the performance of an in-house enzyme-linked immunospot assay (ELISPOT) measuring interferon-γ release by producing T cells in HIV infected individuals in different stages of HIV/AIDS progression, and to compare it with the TST. This study was approved by the Ethics Committee of the Ministry of Health, Senegal.

## Methods

The reported HIV seroprevalence in the general population in Senegal in 2004 was 1.4 % [15]. Approximately 7000 to 8000 new smear positive TB cases are detected each year and the incidence rate was estimated at 132 per 100,000 in 2002 [16].

Recruitment of patients took place at the Infectious Disease Unit and at the Ambulatory Care Centre of Fann Hospital in Dakar. HIV infected individuals over 18 years of age and living in Dakar city, presenting with a diagnosis of HIV infection within the last three months and who had a Karnofsky score of 80 or more were eligible for the study. Subjects with a diagnosis of TB disease within the last 12 months, or with clinical, radiological or mycobacteriological evidence of active TB, as well as those who had received chemoprophylaxis within the last 6 months, were not eligible for the study. Eligible patients were informed of the study protocol and, following discussions in local languages in the presence of at least two members of the research team and a witness, gave individual written consent to participate in the study.

All patients were confidentially interviewed and clinically examined at recruitment. A chest X-ray was performed and blood samples were collected for full blood count, HIV test, CD4/CD8 cell count and EC ELISPOT assay. A TST was administrated by the Mantoux method using 0.1 ml (2 TU) of PPD RT 23 (Statens Serum Institut, Copenhagen). Tests were read by trained study field assistants 48–72 hours later and were measured with a ruler as induration diameters across and along the arm. A mean diameter of greater than 5 mm was taken as a positive test.

HIV serostatus and serotypes were determined using a serial algorithm including an enzyme linked immunosorbent assay (ELISA) (Murex® Ag/Ab, Abbott, Chicago, USA) and an HIV type 1 and type 2 discriminative test (Innolia, Innogenetics, NV, Zwjnaarde, Belgium). Relative counts of CD4^+^ T cells were determined in EDTA-treated peripheral blood by a direct immunofluorescence method [17] using a combination of anti-CD3 peridin chlorophyll protein (PerCP), anti-CD4 fluorescein isothiocyanate (FITC) and anti-CD8 phycoerythrin (PE) on a mobile volumetric flow cytometry instrument, the Cyflow SL blue (Partec GmbH, Münster, Germany).

### The IFN-γ ELISPOT assay

The assay was performed on freshly isolated Peripheral Blood Mononuclear Cells (PBMCs), as previously described [18], predominantly using one pool of 35 15-mer peptides, overlapping by 10 amino-acids (10 µg/ml) and spanning the length of CFP-10 (18 peptides) and ESAT-6 (17 peptides) (Mabtecch AB, Sweden). In a sub-group of patients, the ESAT-6/CFP-10 (EC) ELISPOT was performed with four different peptide pools: pool one contained the first 9 15-mer peptides of ESAT-6 (ES1 to ES9), pool two contained the last 8 peptides of ESAT-6 (ES10 to ES17), pool three was composed of the first 9 peptides of CFP-10 (CFP1 to CFP9) and pool four was composed of the remaining 9 peptides (CFP10 to CFP18). Briefly, 200,000 PBMCs per well were plated directly onto the ELISPOT plate (MAIP, Millipore) in the presence of the peptides, and incubated for 18 hours. PHA (5µg/ml) (Sigma, Missouri, USA) as positive control and media as negative control, were added to duplicate wells. ELISPOT plates were counted using an AID plate reader (Autoimmun Diagnostika, Strassberg, Germany). A positive response to an antigen pool was taken as more than 20 spot forming cells (SFC)/10^6^ PBMC after negative control well SFC subtraction. A failed positive control was defined as less than 100 spots/10^6^ PBMC in the phytohaemagglutinin (PHA) positive control wells [19].

The ELISPOT assay was performed using the combined EC pool in 226 patients. In 45 of them, the test was also performed using the 4 separate pools. Using Bland and Altman's method for assessing agreement between clinical measurements [20], we observed that the combined pool and the maximum of the 4 separate pools did not appear to agree equally through the range of the data, with a tendency for mean difference between the two measurements to rise with increasing response. However, the variances of the two measurements were not significantly different (SD combined pool = 169, SD maximum of 4 separate pools = 152; sd test p = 0.48), which did not suggest that the observed relationship was a genuine trend [21]. We therefore included 59 patients whose EC ELISPOT was performed using the 4 separate pools only. We compared the results of the analysis which included these 59 patients with the results excluding them (data not shown) and found that the results were broadly similar and therefore we report the more complete data.

### Statistical Analysis

All data were double entered into an Access database and checked for errors. Comparisons between the proportions of ELISPOT positive tests were performed using Fisher's exact test. Comparisons between numbers of SFC according to CD4 absolute count were performed using non parametric tests (Kruskal-Wallis), with a significance level of 0.05.

Logistic regression was performed to examine the effect of potential variables on the odds of presenting a positive ELISPOT test response. Odds ratios (OR) and their 95% confidence intervals (CI) were first estimated in a series of univariate analyses. A multivariate model was then constructed, including variables that showed an effect in univariate analysis (p<0·2). BCG scar and gender were forced into the final logistic model. Data were analysed using STATA 7 software (StataCorp LP, USA).

## Results

From October 2003 to March 2005, a total of 298 HIV infected patients were considered for recruitment. From these, 285 (95.6%) were enrolled, 2 (0.6%) refused to participate and 11 (3.7%) presented symptoms and radiological signs for which active TB could not be excluded, and were therefore not included in the study. There were no difference in demographic, clinical and biological characteristics between patients who were included and those who were not (data not shown). The 285 enrolled HIV infected patients were 115 males and 170 females and median age was 37 years (inter-quartile range 30–44) **(** **Table 1** **)**. Clinically, 173 (61%) patients were in CDC class B and 212 (74.4%) in WHO stages I and II. The median CD4 count was 179.5/mm^3^ (range: 2–1553). A BCG scar was present in 207 (72.6%) patients, and 60 (21.4%) had a positive TST (>5 mm) at inclusion. Because HIV infection was recently diagnosed, none of the subjects had received HAART at the time of inclusion.

**Table 1:** Characteristics of patients included in the cohort, with a detail of those responding and those not responding to ELISPOT PHA positive control.

Characteristics	All Patients (n = 285)	PHA<100 SFC/10^6^ (n = 38)	PHA≥100 SFC/10^6^ (n = 247)	p-value*
	Number (%)	Number (%)	Number (%)	
*Gender*				
* - male*	115 (40.4)	14 (36.8)	101 (40·9)	
* - female*	170 (59.6)	24 (63.2)	146 (59·1)	0.72
*Age (years)*				
* - <30*	55 (19.3)	6 (15.8)	49 (19·8)	
* - 30–39*	112 (39.3)	15 (39.5)	97 (39·2)	
* - 40–49*	86 (30.2)	12 (31.6)	74 (30·0)	
* - ≥50*	32 (11.2)	5 (13.2)	27 (10·9)	0.92
*CDC classification*				
* - A*	94 (33.0)	11 (29.0)	83 (33·6)	
* - B*	173 (60.7)	25 (65.8)	148 (59.9)	
* - C*	18 (6.3)	2 (5.3)	16 (6.5)	0.88
*WHO classification*				
* - I*	89 (31.2)	10 (26.3)	79 (32.0)	
* - II*	123 (43.2)	19 (50.0)	104 (42.1)	
* - III*	60 (21.1)	7 (18.4)	53 (21.5)	
* - IV*	13 (4.5)	2 (5.3)	11 (4.4)	0.78
*BCG scar*				
* - Yes*	207 (72.6)	31 (81.6)	176 (71·3)	
* - No*	78 (27.4)	7 (18.4)	71 (28·7)	0.24
*TST (mm)*				
* - 0–5*	224 (78.6)	30 (79.0)	194 (78·5)	0.95
* - >5*	61 (21.4)	8 (21)	53 (21·5)	
*CD4 count (per mm^3^)*				
* - <50*	58 (20.3)	6 (15.8)	52 (21·0)	
* - 50–199*	88 (30.9)	12 (31.6)	76 (30·1)	
* - 200–349*	43 (15.1)	10 (26.3)	33 (13·4)	
* - ≥350*	85 (29.8)	8 (21)	77 (31·2)	
* - undetermined*	11 (3.9)	2 (5·3)	9 (3·6)	0.95
*Haemoglobin level (g/dl)*				
* - <10 g/dl*	141 (49.5)	17 (44.7)	124 (50·2)	
* - ≥10 g/dl*	128 (44.9)	19 (50.0)	109 (44·1)	
* - undetermined*	16 (5.6)	2 (5·3)	14 (5·6)	0.59

* *p* value for difference between PHA<100 and PHA≥100 SFC/10^6^.

**Figure 1:**
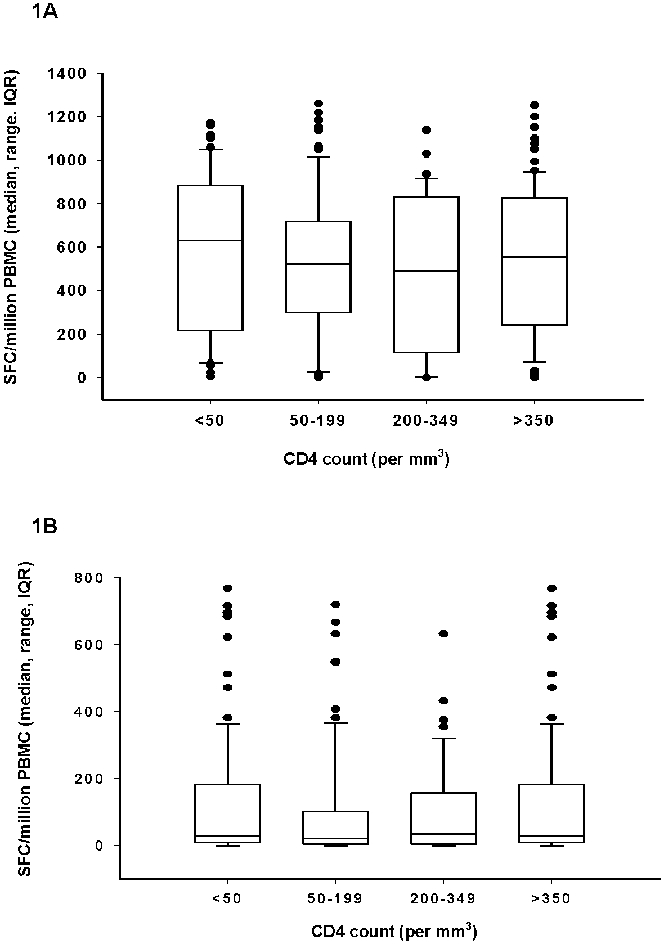
Distribution of responses to PHA and ESAT6/CFP10 ELISPOT assays in HIV patients, by CD4 cell count (boxplot showing median, IQR and range) (n = 285). 1A: Distribution of responses to PHA. 1B: Distribution of responses to ESAT6/CFP10.

The distribution of responses to PHA and to EC ELISPOT according to CD4 count is shown in **Fig 1**. Among the 285 HIV positive patients the median mitogen (PHA) stimulated SFC response was 543 (range 0–1260) and did not vary according to the CD4 cell count (p = 0.518) (**Fig. 1a**). A PHA response inferior to 100 SFC/10^6^ PBMC was however found in 38 (13.3%) patients, reflecting a failed positive control response. These subjects were therefore excluded from further analysis. Of note, these subjects had similar demographic and clinical characteristics as positive PHA responders **(** **Table 1** **)**.

Among the 247 remaining HIV positive patients, the mean SFC/10^6^ PBMC produced in response to stimulation with EC antigens was 103 (standard deviation: 186) and the median 22 (range: 0–1247). Using the threshold of 20 SFC/10^6^ PBMC, 125 of the 247 (50.6%) subjects had a positive EC ELISPOT response, while the TST was positive in 53 (21.5%) individuals only. Concordance between EC ELISPOT and TST was observed in 151 patients (61.1%) (kappa: 0.23). However, 84 (34%) subjects were EC-ELISPOT positive and TST negative, while 12 (4.9%) were TST positive and EC-ELISPOT negative. The relative proportion of positive responses with each test did not change significantly when using cut-offs of 10 SFC/10^6^ or 30 SFC/10^6^ (data not shown).

**Figure 2:**
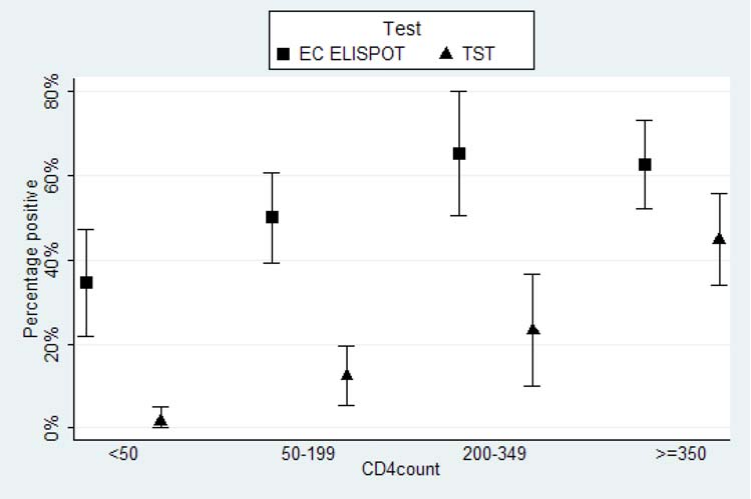
Percentage of positive responders to ESAT6/CFP10 ELISPOT and to TST, by CD4 cell count (n = 247).

The mean total lymphocyte count was similar in HIV infected patients who showed a positive response to EC-ELISPOT (38%) and those who did not (37%), and the median EC stimulated SFC did not vary according to the CD4 cell count (p = 0.3) (**Fig. 1b**). However, the proportion of positive responses to the EC ELISPOT assay decreased with decreasing CD4 counts (test for trend: p = 0.0012) **(** **Fig 2** **)**, and for all categories of CD4 count, the proportion of responders to EC ELISPOT was consistently higher than the proportion of TST responders. Of note, the proportion of patients with a BCG scar was distributed evenly across the CD4 count categories.

We examined the effect of given demographic and clinical factors on the likelihood of developing a positive response to the EC ELISPOT assay as well as to the TST **(** **Table 2** **)**. This analysis confirms the effect of CD4 cell count on the risk of positive EC ELISPOT and TST responses (p = 0.016 and p<0.0001 respectively). Positive responses to EC ELISPOT were associated with age, conversely to TST positivity, but not with sex or family history of TB. EC ELISPOT positivity was associated with HIV1 infection, but not with the clinical severity of disease, as assessed through CDC classification. As expected, the presence of a BCG scar had an effect on TST positivity, but not on EC ELISPOT positivity.

**Table 2:** Analysis of the determinants of positive response to the ESAT6/CFP10 ELISPOT assay and to the TST (n = 247).

Variables	N	TST positive response				EC Elispot positive response			
		Univariate OR [95% CI]	p	Adjusted OR [95% CI]	p	Univariate OR [95% CI]	p	Adjusted OR [95% CI]	p
*CD4 class*									
* - <50*	52	1		1		1		1	
* - 50–199*	76	8.1 [1.0–64.9]		8.5 [1.0–69]		1.7 [0.8–3.5]		1.5 [0.7–3.2]	
* - 200–349*	33	17.3 [2.1–141]		14.8 [1.7–129]		2.9 [1.2–7.2]		2.7 [1.1–7.1]	
* - ≥350*	77	46.1 [6.1–348]		36.6 [4.6–288]		3.1 [1.5–6.5]		3.2 [1.5–7.0]	
* - undetermined*	9	5.7 [0.3–98]	0.0001	5.5 [0.3–97]	0.0001	0.9 [0.2–4.2]	0.018	0.7 [0.1–3.8]	0.016
*HIV type*									
*- HIV 1 - dually infected*	216	1		-	-	1		1	0.02
*- HIV 2*	32	0.64 [0.3–1.4]	0.27			0.4 [0.2–0.9]	0.03	0.3 [0.1–0.8]	
*CDC class*									
*- A*	83	1		1		1		-	-
*- B or C*	164	1.9 [1.1–3.3]	0.03	1.11 [0.5–2.2]	0.5	1.2 [0.7–2.0]	0.59		
*Age (years)*									
*- <30*	49	1		1		1		1	
*- 30–39*	97	1.2 [0.5–2.6]		1.0 [0.4–2.7]		0.9 [0.5–1.8]		0.9 [0.4–1.9]	
*- 40–49*	74	1.3 [0.5–3.0]		1.3 [0.4–3.7]		0.7 [0.3–1.5]		0.8 [0.4–1.8]	
*- ≥50*	27	1.7 [0.6–4.9]	0.74	1.7 [0.5–6.1]	0.78	5.0 [1.5–16.9]	0.016	4.3 [1.3–13.6]	0.024
*Gender*									
*- Male*	101	1		1		1		1	
*- Female*	146	0.7 [0.4–1.3]	0.32	0.8 [0.5–1.3]		0.8 [0.5–1.3]	0.45	0.7 [0.4–1.3]	0.26
*BCG scar*									
*- No*	71	1		1		1		1	
*- Yes*	176	3.6 [1.5–8.3]	0.003	3.3 [1.2–8.6]	0.01	1.1 [0.6–2.0]	0.41	1.1 [0.6–2.0]	0.75
*Family history of TB*									
*- No*	239	1		-	-	1		-	-
*- Yes*	8	2.3 [0.5–9.8]	0.26			2.7 [0.5–20.1]	0.27		

## Discussion

The identification and treatment of latent TB infection (LTBI) in HIV positive individuals is one of the main recommendations developed by the World Health Organisation in order to reduce morbidity and mortality in HIV patients living in high TB prevalence areas [5]. Tuberculin skin testing has been shown to be highly inadequate for identifying LTBI in HIV infected individuals, as the anergy to skin testing may lead to false negative results [7]. More sensitive and specific diagnostic tools are therefore urgently required [22], [23]. We report here the results from a study comparing an in-house ESAT-6/CFP-10 (EC) assay with TST for the diagnosis of LTBI in an HIV infected cohort in a TB endemic country. Our study showed that, similar to findings in HIV negative cohorts [11], [24], [25], the EC-ELISPOT assay appears more sensitive than the TST in HIV infected individuals, but this sensitivity appears impaired in those with severe immuno-suppression.

The sensitivity and specificity of a new diagnostic test is usually assessed through comparison with a gold standard diagnostic assay. While the gold standard for TB disease is the positive culture of *M. tuberculosis*, there is no such standard for TB infection. However, given that TB disease is highly prevalent in HIV patients [2] and that the TST has a high false negative rate for the diagnosis of TB disease in these patients, the increased frequency of positive responses to the EC-ELISPOT in HIV patients when compared to the TST is likely to reflect an increased sensitivity of the former. To our knowledge, this is the largest study reporting such results in a high TB prevalent country affected with HIV infection.

The recent breakthrough brought about by the development of TB specific T-cell based IGRA has raised high hopes for an improved diagnosis of LTBI, with an expectedly wide impact on TB control. IGRA do indeed carry several advantages over the century old TST [26]: testing requires only 1 patient visit and as these are ex-vivo tests, the risk for adverse effects is reduced and potential boosting is eliminated when testing is repeated. However, IGRAs are costly, impose blood drawing, and necessitate appropriately equipped laboratory. In addition, samples need to be carefully handled to maintain viability of lymphocytes. Current evidence suggests that IGRA have higher specificity than the TST, better correlation with surrogate markers of exposure to *M. tuberculosis* in low-incidence settings, and less cross-reactivity to BCG vaccination than the TST [26], [27].

In HIV infected persons, there is a concern that, similarly to the TST [28], the performance of the test could be impaired due to anergy associated with decreasing CD4 cell counts resulting in false negative ELISPOT responses [26]. The performance of whole-blood cytokine assays for the diagnosis of LTBI in HIV positive versus HIV-negative patients has previously been assessed in two small studies in Africa, with conflicting results. In a study in Uganda, Elliot *et al* found that the IFN-γ response to PPD and CFP-10 antigens was strongly impaired among HIV positive compared to HIV negative subjects [13]. In contrast, in a study conducted in Zambia, the sensitivity of the ELISPOT assay (using PPD and ESAT-6 or CFP-10 antigens) was only slightly reduced in HIV positive patients (90%), as opposed to HIV negative patients (100%) [14].

There are very few reports on the performance of IGRAs according to the degree of immuno-suppression. Many laboratories use PHA stimulation as a positive control in cellular assays, such as the ELISPOT. To assess the utility of IGRAs in immuno-suppressed individuals, authors have examined whether responses to PHA decrease with CD4 T cell count. In a study assessing response to *M. tuberculosis* specific antigens among 590 HIV positive patients in Denmark using the Quantiferon-TB In Tube test, the proportion of patients with indeterminate test results due to low IFN-γ production following PHA stimulation was significantly higher in patients with low CD4 count (<100 cells/ml) compared to those with high CD4 cell count (> 100 cells/ml), and the median IFN-γ release was significantly lower in those persons with a low CD4 cell count compared with patients with a high CD4 cell count [29]. However, we and others have shown that, when using the ELISPOT assay, the PHA response did not vary with CD4 cell count [19], [30]. In the ELISPOT assay, PBMCs are isolated and counted, and known numbers of cells are added into each well. Therefore, as the CD4 cell count falls, more PBMCs are added into the ELISPOT assay to compensate this fall, and the PHA response is maintained. In our study, in all CD4 count classes, the EC ELISPOT showed consistently improved sensitivity over the TST. However, despite a maintained PHA response, the proportion of patients with a positive response to the EC ELISPOT decreased with the CD4 cell count, and this was particularly marked in severely immuno-suppressed individuals

The low proportion of positive responses to the EC-ELISPOT in HIV infected person with CD4 cell counts lower than 200/mm^3^, and particularly among those with less than 50/mm^3^, could either reflect a smaller proportion of patients being infected with *M. tuberculosis* in that group of subjects, or more likely, the suppression of the antigen-specific *M. tuberculosis* T cell response with the progression of HIV infection, as has been shown by Sutherland *et al*. in chronic HIV-1 infected persons [31]. Altogether, our results confirm the findings by Chapman *et al* of a higher sensitivity of the EC-ELISPOT assay compared to TST for the diagnosis of LTBI in HIV infected individuals [14], but also suggest that the sensitivity of this test be dependant on HIV progression. Thus, IGRA assays appear very useful for the diagnosis of LTBI in HIV infected individuals, with an increased performance compared with the TST, but in severely immuno-suppressed individuals, the test may be impaired by T-cell anergy, warranting a careful interpretation of negative results in these patients.

The multivariate analysis showed that the positive response to EC-ELISPOT assay was associated with age, CD4 count and HIV-1 strain. The EC-ELISPOT was more likely to be positive in individuals aged more than 50 years, which may reflect repeated exposure to TB infection that would occur during the lifetime in an endemic setting. It has previously been shown that TST positivity increased with age in an HIV negative cohort [32]. The lack of association between TST and age in our study may be due to the strong impact of HIV infection on TST and the high level of TST anergy observed. The moderate effect seen in the older age group is in favour of increased sensitivity of the EC ELISPOT in comparison with the TST in this HIV infected population.

The difference of effect observed with the HIV type may be related to epidemiological and biological differences. While HIV-1 is found across the globe, HIV-2 is confined mainly to West Africa. Rates of disease progression are, however, significantly lower in HIV-2 infection when compared to HIV-1 [33] and as TB disease is associated with increased HIV progression it is not surprising that in our study TB infection is associated with HIV-1 strain.

In conclusion, this study showed that the EC IFN-γ release assay is likely to retain its validity for the diagnosis of LTBI among HIV positive patients, with the caveat that its sensitivity decreases with advanced immuno-suppression. Establishing the critical level of immuno-suppression under which the performance of the IGRA may be improved (i.e. determining the critical CD4 cut-off) is now a priority. This will enable this new T cell based test to contribute usefully to the diagnosis of LTBI in HIV infected persons and would usefully contribute to TB control by improving the direction of preventive therapy among HIV positive patients.
